# Supplementary material for: A Genome-Wide Survey of Genetic Variation in Gorillas Using Reduced Representation Sequencing
Source: PLoS One. 2013 Jun 4;8(6):e65066. doi: 10.1371/journal.pone.0065066 (PMC3672199; doi:10.1371/journal.pone.0065066)
Supplement: File S1 — Gorilla family trees. Information on the geographical origin of the sampled gorillas are based on the capture locations of the gorillas themselves or (in the case of captive-born individuals) the capture locations of their wild-born ancestors. (PDF) [file pone.0065066.s001.pdf]

Supplementary information 1: Gorilla family trees. Information on the geographical origin of the sampled gorillas are based on the capture locations of the gorillas themselves or (in the case of captive-born individuals) the capture locations of their wild-born ancestors.

1) Snowflake, AKA Floquet (#0281)
Born in the wild (~1962)
Captured in Equatorial Guinea (source: Jonch, 1968)

2) Guy (#0005)
Born in the wild (~1946)
Captured in Cameroon (then French Cameroons) (source: Natural History Museum)

3) Mukisi (#9912)
Born in the wild (~1957)
Captured in the Walikale region, Democratic Republic of Congo (source: Jensen-Seaman & Kidd, 2001)

4) Effie's family:

Effie (#1287)
Born in Copenhagen Zoo (1993)

Samson (#0492)
Born in the wild (~1972)
Capture location unknown

Salome (#0530)
Born in the wild (~1972)
Captured in Cameroon
(source: Gorilla studbook)

5) BiKira's family:

BiKira (#1352)
Born in Amsterdam Zoo (1995)

Tembo (#0185)
Born in the wild (~1961)
Captured in Cameroon
(source: Gorilla studbook)

Dafina (#0972)
Born in Stuttgart Zoo (1987)

Banjo (#0255)
Born in the wild (~1965)
Captured in Cameroon
(source: Gorilla studbook)

Dina (#0477)
Born in the wild (~1970)
Capture location unknown

6) Kaja's family:

Kaja (#0700)
Born in Howletts Zoo Park (1979)

Kisoro (#0184)
Born in the wild (~1961)
Capture location unknown

Juju (#0372)
Born in the wild (~1962)
Captured in Cameroon (then Southern Cameroons)
(source: Aspinall, 1976)

7) Victoria's family:

Victoria (#9919)
Born in Antwerp Zoo (1968)

Kisubi (#9908)
Born in the wild (~1955)
Captured in the Walikale region,
Democratic Republic of Congo
(source: Hofman)

Quivu (#9917)
Born in the wild (~1961)
Captured in the Walikale region,
Democratic Republic of Congo
(source: Hofman)

8) Murphy's family

Murphy (#0684)
Born in Omaha's Henry Doorly Zoo (1978)

Casey I (#0083)
Born in the wild (~1956)
Captured in West Africa
(source: Gorilla studbook)

Bridgette (#0226)
Born in the wild (~1961)
Captured in West Africa
(source: Gorilla studbook)

9) Kamilah's family:

Kamilah (#0661)
Born in San Diego Zoo (1977)

Pete (#0384)
Born in the wild (~1967)
Captured in Cameroon
(source: Gorilla studbook)

Nina (#0330)
Born in the wild (~1968)
Capture location unknown

10) Kesho's family:

Kesho (#1521)
Born in Dublin Zoo (1999)

Horst, AKA Harry (#0935)
Born in Frankfurt Zoo (1986)

Xerna, AKA Lena (#0803)
Born in Rotterdam Zoo (1982)

Matze (#0337)
Born in the wild (~1957)
Capture location unknown

Dorette (#0276)
Born in the wild (~1965)
Capture location unknown

Ernst (#0566)
Born in the wild (~1972)
Captured in Cameroon
(source: Gorilla studbook)

Xara (#0402)
Born in the wild (~1969)
Captured in Congo
(source: Gorilla studbook)

11) Ruby's family :

Ruby, AKA B'windi (#1437)
Born in Arnhem Zoo (1997)

Gorgo (#0766)
Born in Krefeld Zoo (1981)

Bebe (#0860)
Born in the wild (~1979)
Captured in Cameroon
(source: Gorilla studbook)

Massa (#0578)
Born in the wild (~1971)
Capture location unknown

Boma (#0588)
Born in the wild (~1973)
Captured in Cameroon
(Source: Gorilla studbook)

12) Matadi's family:

Matadi (#1745)
Born in Twycross Zoo (2003)

Sekondi, AKA TI (#1085)
Born in Edinburgh Zoo (1989)

Ozala (#1327)
Born in Twycross Zoo (1994)

Sam Sam (#0529)
Born in the wild (~1972)
Captured in Cameroon
(source: Gorilla studbook)

Yinka (#0469)
Born in the wild (~1972)
Captured in Cameroon
(source: Gorilla studbook)

Mamfe (#0497)
Born in Jersey Island Zoo
(1973)

Biddy (#0635)
Born in the wild
(~1973)
Capture location
unknown

Jambo (#0111)
Born in Basel Zoo (1961)

N'Pongo (#0090)
Born in the wild
Captured in Cameroon
(source: Gorilla studbook)

Stefi, AKA Christopher (#0038)
Born in the wild (~1949)
Captured in Cameroon
(source: Gorilla studbook)

Achilla (#0008)
Born in the wild (~1947)
Captured in Cameroon
(source: Gorilla studbook)

13) Fubu's family:

Fubu (#1742)
Born in Howletts Zoo Park (2003)

Kifu (#0954)
Born in Howletts Zoo Park (1987)

Bamilla (#0960)
Born in Howletts Zoo Park (1987)

Kijo (#0577)
Born in Howletts Zoo Park
(1975)

Founa (#0457)
Born in the wild (~1971)
Captured in Congo
(source: Aspinall, 1976)

Bitam (#0458)
Born in the wild (~1971)
Captured in Congo
(source: Aspinall, 1976)

Killa Killa (#0683)
Born in Howletts Zoo Park
(1978)

Kisoro (#0184)
Born in the wild (~1961)
Capture location unknown

Juju (#0372)
Born in the wild (~1962)
Captured in Cameroon
(then Southern Cameroons)
(source: Aspinall, 1976)

Kisoro (#0184)
Born in the wild (~1961)
Capture location unknown

Mouila (#0371)
Born in the wild
(~1960)
Captured in
Cameroon
(then Southern
Cameroons)
(source: Aspinall,
1976)

14) EB(JC): The sample from EB(JC) was purchased from the European Collection of Cell Cultures (ECACC), and we do not have any information on its origin.

Sources for capture locations:

- Aspinall, J. (1976). The Best of Friends. London: MacMillan
- Hofman, S. Curator of Mammals, Antwerp Zoo, personal communication
- International Gorilla Studbook (2001, 2006 and 2010), published by Frankfurt Zoo.
- Jonch, A. (1968). The white Lowland gorilla Gorilla g. gorilla at Barcelona Zoo. International Zoo Yearbook, 8(1): 196-197.
- Jensen-Seaman, M.I. and Kidd, K.K. (2001). Mitochondrial DNA variation and biogeography of eastern gorillas. Molecular Ecology, 10: 2241–2247
- Natural History Museum website (07/12/2012) http://www.nhm.ac.uk/nature-online/collections-at-the-museum/museum-treasures/guy-the-gorilla/index.html
