# Supplementary material for: A Genome-Wide Survey of Genetic Variation in Gorillas Using Reduced Representation Sequencing
Source: PLoS One. 2013 Jun 4;8(6):e65066. doi: 10.1371/journal.pone.0065066 (PMC3672199; doi:10.1371/journal.pone.0065066)
Supplement: File S2 — Supplementary Figures S1–S6 and Table S1. (PDF) [file pone.0065066.s002.pdf]

A genome-wide survey of genetic variation in gorillas using reduced representation sequencing

Supplementary figures and tables

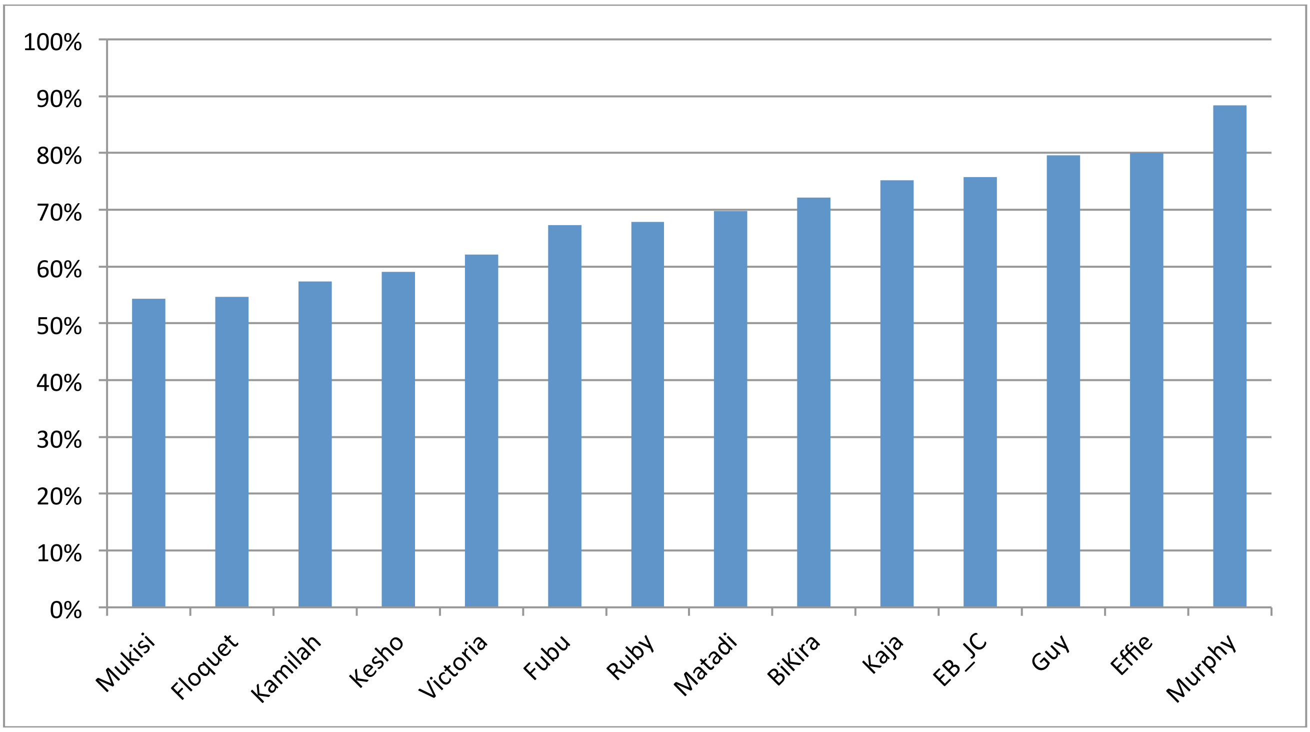

Figure S1: Percentage of target *Alu*I fragments sites (150-250) bp covered in the 14 gorillas sequenced.

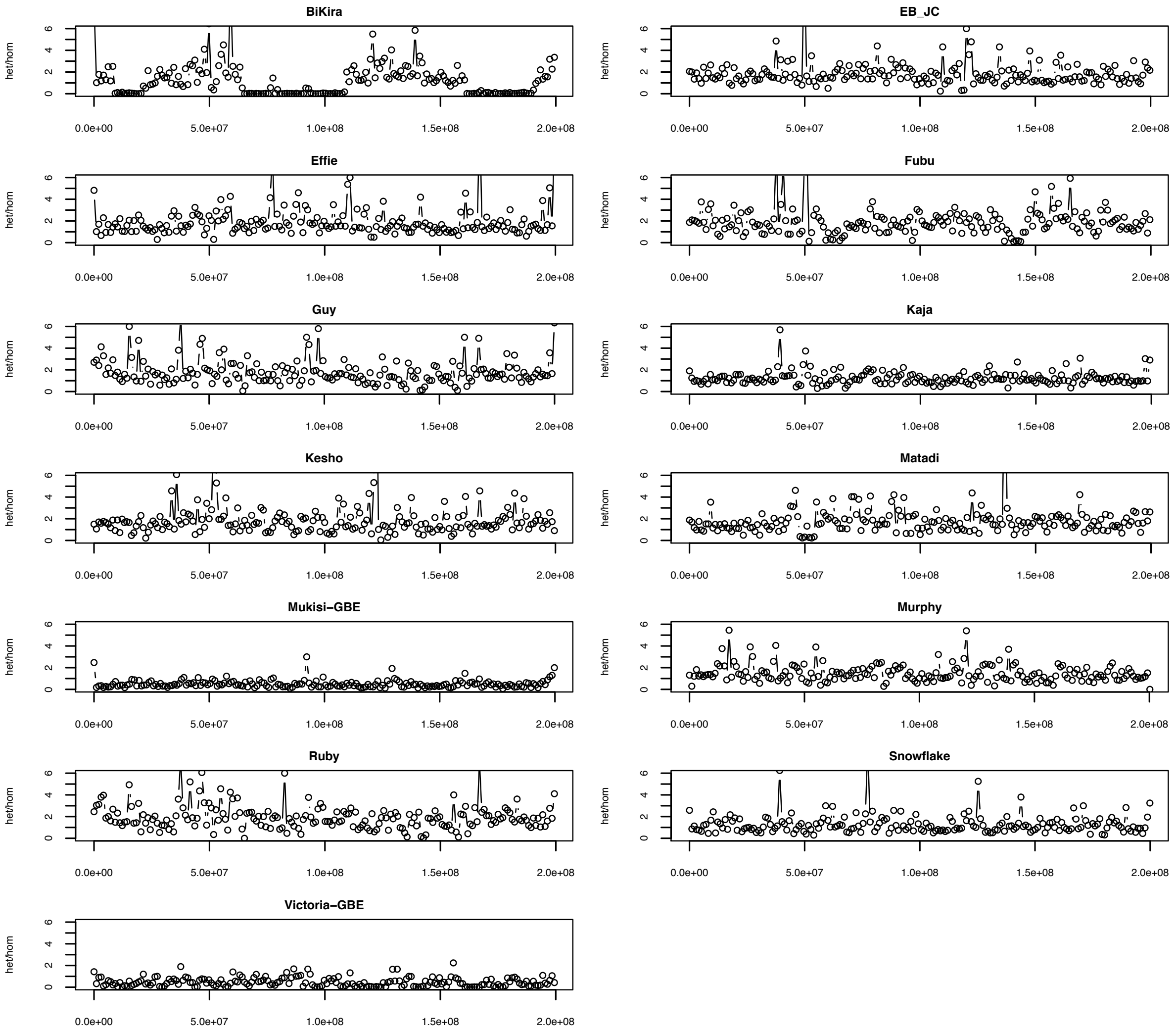

Figure S2: Ratio of heterozygous to homozygous variant sites in 1 Mbp bins along Chromosome 3 for 13 gorillas. Note the extended tracts of homozygosity in BiKira. Other chromosomes (not shown) display a similar pattern.

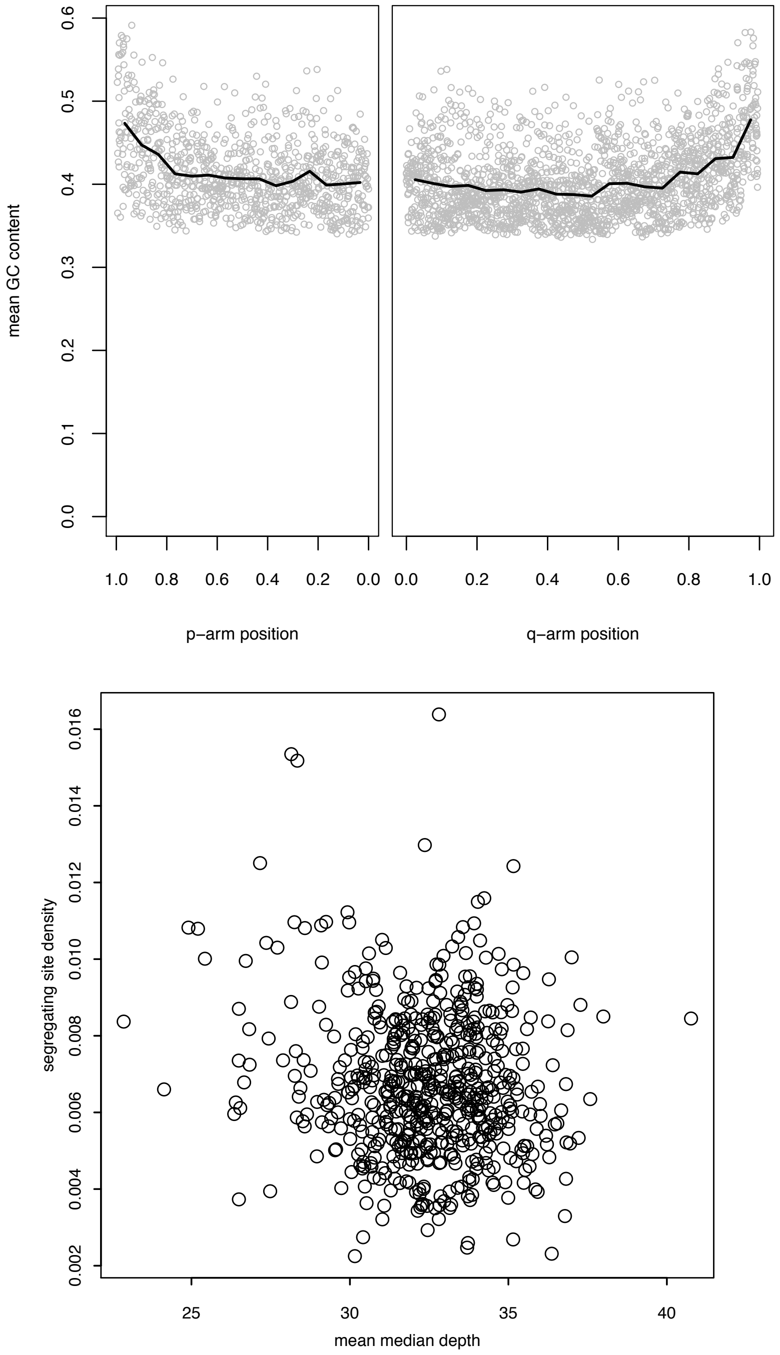

Figure S3: GC content does not explain the pattern of heterozygosity seen in Figure 2. *Upper panel*: GC does increase on average towards the telomeres, but the same is not true in pericentromeric regions. *Lower panel:* there is no significant correlation between the density of segregating sites in 1 Mbp regions genome wide and sequence depth (where the latter is represented by the mean median depth of all samples at each base in each region).

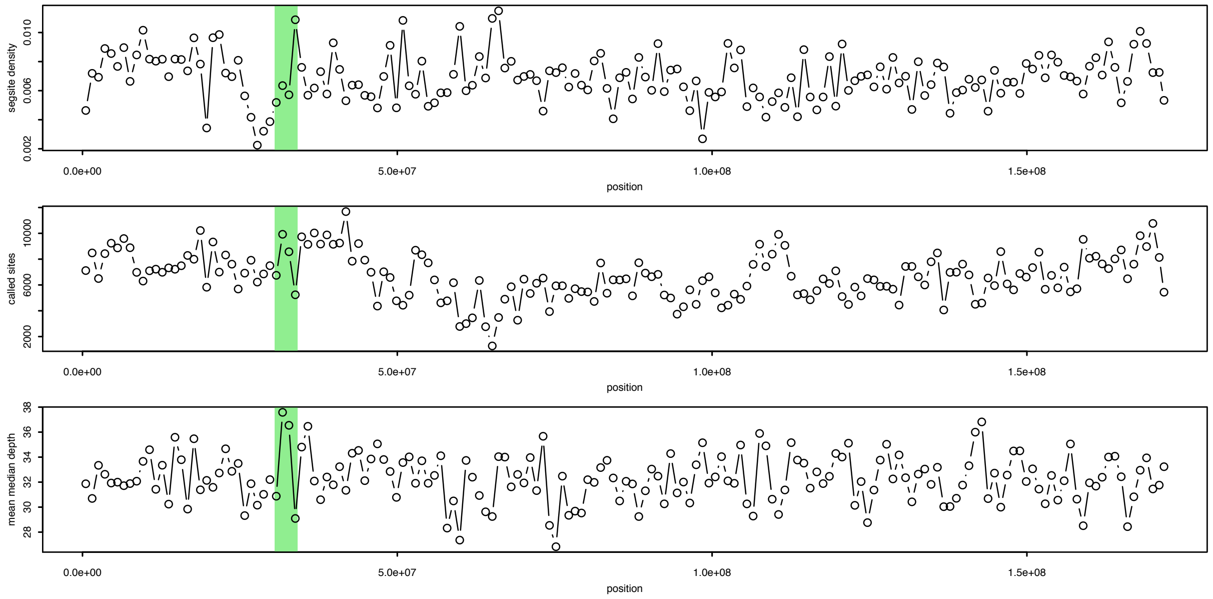

Figure S4: Western lowland gorilla polymorphism rate, number of called sites and mean median depth along Chromosome 6. Each point represents a 1 Mbp bin. The green shaded region indicates the location of the MHC.

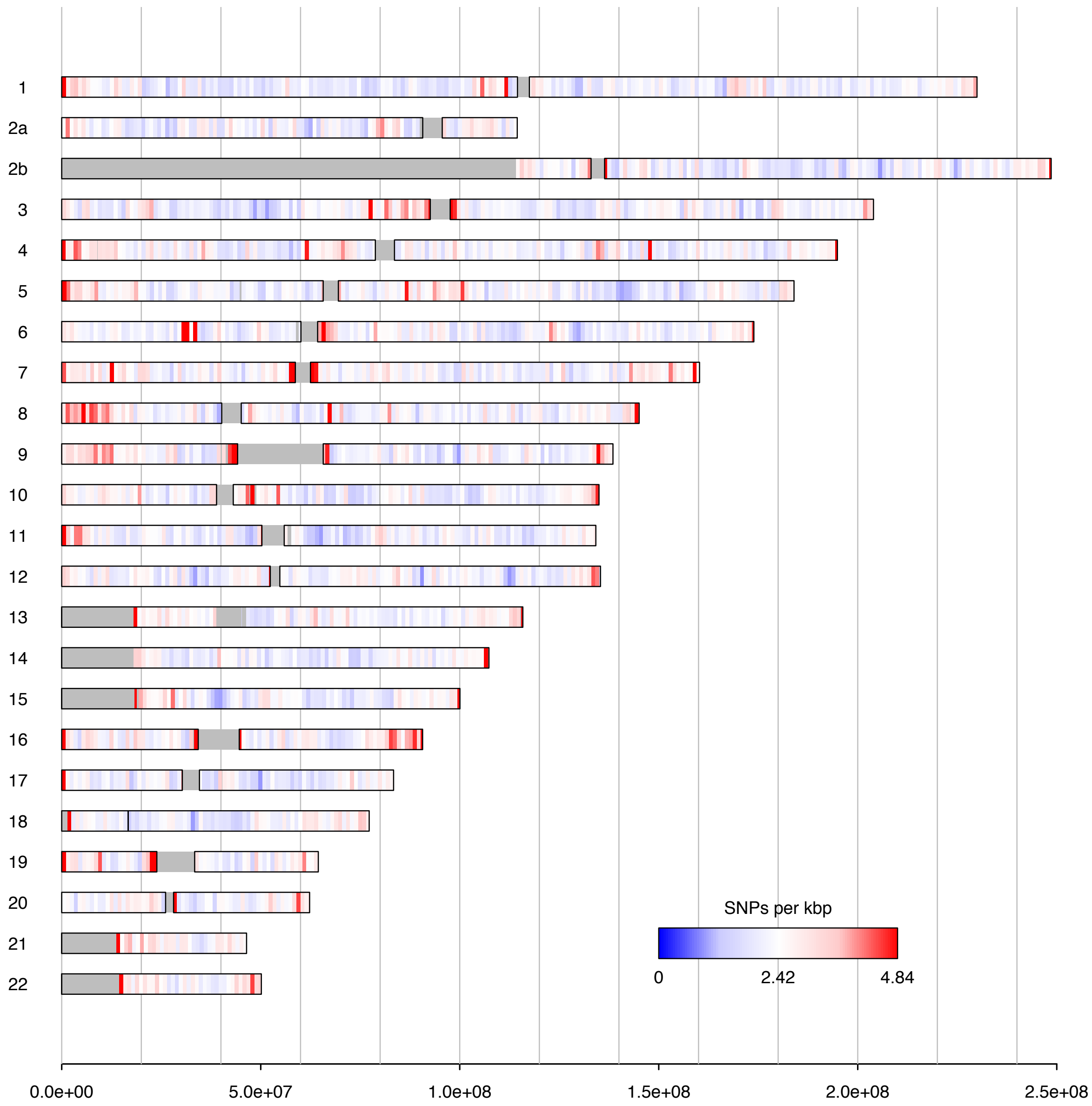

Figure S5: Density of segregating sites in ten western chimpanzees (*Pan troglodytes verus*). Segregating sites are based on genome-wide sequencing data from [20]. Sites passing quality and depth filtering thresholds in all ten samples were binned in 1 Mb bins and the density of segregating sites calculated. Resulting densities are plotted on an ideogram, with the scale expressed as number of sites per kbp. Note the prominent signal of increased diversity at the MHC locus on 6p.

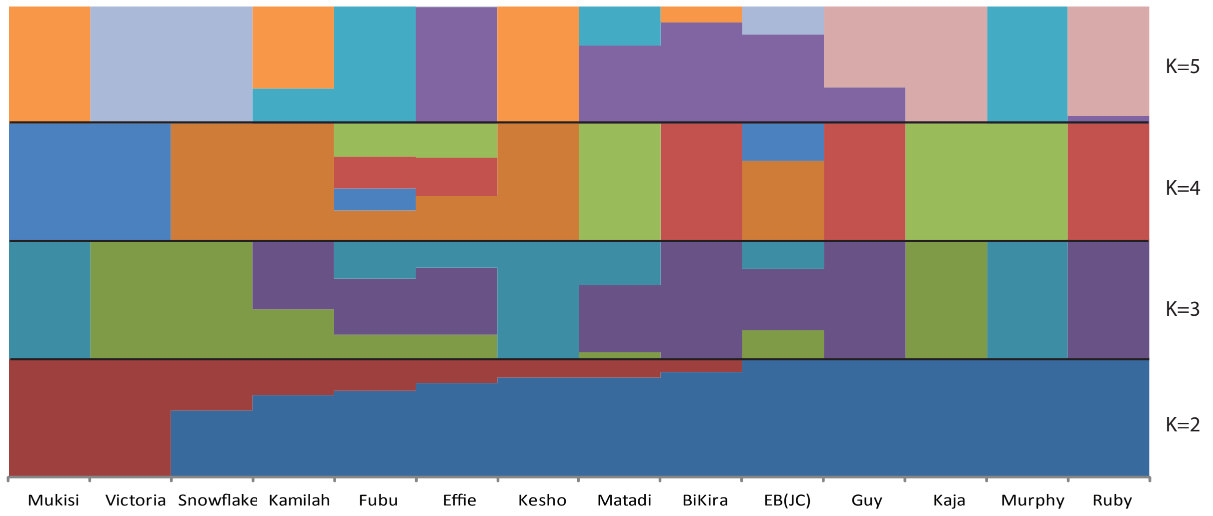

Figure S6: ADMIXTURE plot of eastern lowland and western lowland gorillas. Each gorilla is represented by a single vertical bar and the proportion of ancestry is displayed in different colours. For K=2, red represents the eastern component and blue the western component; this was estimated to be the optimum number of clusters based on a cross-validation procedure. The estimation of individual ancestries based on K=3, 4 and 5 ancestral populations are also shown.

Table S1: SNPs called per chromosome. SNPs in repeat masked regions or at sites where the coverage depth was less than 10 or more than 100 in at least one individual were excluded.

Chr	SNPs	Transitions	Transversions	Ts/Tv ratio
1	248308	168454	79854	2.109524883
2a	128033	86992	41041	2.119636461
2b	139088	94870	44218	2.145506355
3	196184	131977	64207	2.055492392
4	200278	136286	63992	2.129734967
5	175019	118626	56393	2.103558952
6	186986	127638	59348	2.150670621
7	179279	121923	57356	2.125723551
8	159431	108019	51412	2.101046448
9	139052	94840	44212	2.145118972
10	161205	111192	50013	2.223261952
11	145754	98502	47252	2.084610175
12	128856	87842	41014	2.141756473
13	111075	76125	34950	2.178111588
14	94184	64154	30030	2.136330336
15	95229	64728	30501	2.122159929
16	97558	65957	31601	2.087180785
17	107183	73572	33611	2.188926244
18	89645	61433	28212	2.177548561
19	54699	37230	17469	2.131203847
20	71004	48826	22178	2.201551087
21	46855	32690	14165	2.307800918
22	51765	36461	15304	2.382449033
**TOTAL**	**3006670**	**2048337**	**958333**	**2.137395874**
